# Supplementary material for: Patterns of Intron Gain and Loss in Fungi
Source: PLoS Biol. 2004 Nov 30;2(12):e422. doi: 10.1371/journal.pbio.0020422 (PMC532390; doi:10.1371/journal.pbio.0020422)
Supplement: Table S1 — Also available at http://genes.mit.edu/NielsenEtAl/. (4.3 MB ZIP). [file pbio.0020422.st001.zip › NielsenEtAl/html/415.html]

AN4235.1.NCU08609.1.MG04527.1.FG01138.1


```
 CLUSTAL W (1.82) Multiple Sequence Alignments - Introns Inserted


Sequence 1: NCU08609.1	345 aa
Sequence 2: FG01138.1	331 aa
Sequence 3: MG04527.1	347 aa
Sequence 4: AN4235.1	308 aa
Alignment Length: 364 aa
Number Identitical Residues: 123 aa
Alignment Score (without introns) 6981


MG04527.1 	-------MQHGVR---SILPLVLTACLFLG-AANASVGDRLPEFRECVQ0VCLHENCEAG
NCU08609.1	MKFNVTSKPGGAPSFLSIQQLLLLGFVALATPVAASIGDRLPEFQECIR0VCERENCGPD
FG01138.1 	-----MATQRGRP-WARIISLTVL-VLTFAITVDASTGDRLPEFKDCLK0VCNAENCAPN
AN4235.1  	----MIRSPAVRKVGYCFLFLLLASCVQES---SASLGDHLPDFKSCVK0ICEAENCQDG
          	                 :  * : . :  .    ** **:**:*:.*:: :*  ***  .

MG04527.1 	TLAHKVETP1LIN~RLLLWTCPAECDYTCQHIITSDRIESG-----QPVVQFHGKWPFYR
NCU08609.1	A-EHQTPIP1LHR~RLLLWSCPSECDYTCQHLTTSSRLSQSPPPLPHPVVQYHGKWPFIR
FG01138.1 	--KPQTPIP1VLH~RLLLWNCASECDYACQHIVTGQRMATG-----LSVEQFYGKWPFYR
AN4235.1  	D-------S~AIH1RRLARDPP---------MLS-------------PVVQFHGKWPFRR
          	        .   . * *    .         : :             .* *::***** *

MG04527.1 	VLGMQEPFSVIFSAGNLYAHLLGFR-WLRRHIPESYPLRKYYVGFSFAGVASWLFSIIFH
NCU08609.1	FLGMQEPLSVLFSLGNFWAHYQGLYTKILPNIPPSYPLRKWYILLSYVGMASWFFSAVFH
FG01138.1 	FLGMQEPFSVLFSLGNLWAHWYGLKTMDQARIPKSYSMRIFYDWLAYIGIASWTFSSIFH
AN4235.1  	ILGMQELFSVIFSGLNFLAHWYGMA-RLREMTPSWHPLQKYYIAFGYSGLAAWTFSMLFH
          	.***** :**:**  *: **  *:        *  :.:: :*  :.: *:*:* ** :**

MG04527.1 	TRDTRATEQLDYFAAGASVLYGLYLAVIRIFRLDRPGSTDGGKTPTGTASSSSSISSSTP
NCU08609.1	TRDFPVTEQLDYFAAGANVLYGLYYTVVRIFRLDKKD------TPRRES-----------
FG01138.1 	TRDFHVTEELDYFAAGASVLYGLYYTVVRVFRLDKR-------TPRRRT-----------
AN4235.1  	ARDFPLTEKLDYFGAGASVLYGLYLATVRIFRLDKE-------QPRYRP-----------
          	:**   **:****.***.****** :.:*:****:         *   .           

MG04527.1 	RAIRLWTAACLVAYGCHVAYLKLVRWDYGYNMAANVCVGLAQNVLWSAFSYRKYTREGRT
NCU08609.1	-LLRLWTALCILMYVAHVTYLKMWAWDYTYNMAANVAVGAIQNLLWSWYSWTRYREQKKG
FG01138.1 	-TLRLWSLLCASLFLSHVSYLKFVRWDYTYNMAANVAAGIVQHVLWSWFSFNRYRESRRI
AN4235.1  	TLRRLWTTVCILLYTIHVCYLSFWSWDYTYNMIANIVVGMIQNTLWICFSVVRYQKTGKT
          	   ***:  *   :  ** **.:  *** *** **: .*  *: **  :*  :* .  : 

MG04527.1 	WATYPGLAVAWIMLAMSLELFDFPPLWGALDAHALWHLGTIAPAVLWYS2FLVKDAQDDM
NCU08609.1	WAAWPGIVVAWVLVAMSLELLDFPPLWGSVDAHSLWHAGTIVPTIIWYN2FLVKDAQDDM
FG01138.1 	WAAWPGFVVAWIIFAMSMELFDFPPWLGCIDAHSLWHLMTIGPTILWYN2FLVKDAQDDM
AN4235.1  	WTLWPALIVVWIILAMSLELLDFPPWYALIDAHSLWHLGTVIPCAWWYL2YLVKDIQDDV
          	*: :*.: *.*::.***:**:****  . :***:***  *: *   **  :**** ***:

MG04527.1 	ASAERFKD
NCU08609.1	ARSERLKS
FG01138.1 	ARSQRLKL
AN4235.1  	AG-ERLKA
          	*  :*:*
```
